# Pharmacokinetics of cannabichromene in a medical cannabis product also containing cannabidiol and Δ^9^-tetrahydrocannabinol: a pilot study

**DOI:** 10.1007/s00228-021-03232-8

**Published:** 2021-10-18

**Authors:** Erica N. Peters, Laura MacNair, Irina Mosesova, Uwe Christians, Cristina Sempio, Jost Klawitter, M. Hunter Land, Mark A. Ware, Cynthia Turcotte, Marcel O. Bonn-Miller

**Affiliations:** 1 Canopy Growth Corporation, One Hershey Drive, Smiths Falls, ON Canada; 2 iC42 Clinical Research and Development, Department of Anesthesiology, University of Colorado Anschutz Medical Campus, Aurora, CO USA

**Keywords:** Cannabichromene, Phytocannabinoid, Pharmacokinetics, Cannabis

## Abstract

**Purpose:**

Cannabichromene (CBC) is a phytocannabinoid commonly found in cannabis, yet its acute post-dose pharmacokinetics (PK) have not been examined in humans. This is a secondary data analysis from a trial investigating Spectrum Yellow oil, an oral cannabis product used for medical purposes that contained 20 mg cannabidiol (CBD), 0.9 mg Δ^9^-tetrahydrocannabinol (THC), and 1.1 mg CBC, per 1 mL of oil.

**Methods:**

Participants (*N* = 43) were randomized to one of 5 groups: 120 mg CBD, 5.4 mg THC, and 6.6 mg CBC daily; 240 mg CBD, 10.8 mg THC, and 13.2 mg CBC daily; 360 mg CBD, 16.2 mg THC, and 19.8 mg CBC daily; 480 mg CBD, 21.6 mg THC, and 26.4 mg CBC daily; or placebo. Study medication was administered every 12 h for 7 days. Plasma CBC concentrations were analyzed by a validated two-dimensional high-performance liquid chromatography–tandem mass spectrometry assay.

**Results:**

After a single dose and after the final dose, the *C*_max_ of CBC increased by 1.3–1.8-fold for each twofold increase in dose; the *t*_max_ range was 1.6–4.3 h. Based on the ratio of administered CBD, THC, and CBC to the plasma concentration, the dose of CBD was 18 times higher than the dose of CBC, yet the AUC_0–*t*_ of CBD was only 6.6–9.8-fold higher than the AUC_0–*t*_ of CBC; the dose of THC was similar to the dose of CBC, yet THC was quantifiable in fewer plasma samples than was CBC.

**Conclusions:**

CBC may have preferential absorption over CBD and THC when administered together.

*Trial Registration*: Australian New Zealand Clinical Trials Registry #ACTRN12619001450101, registered 18 October 2019.

**Supplementary information:**

The online version contains supplementary material available at 10.1007/s00228-021-03232-8.

## Introduction

Medical use of cannabis to treat a variety of therapeutic indications is growing worldwide [1]. Most medical cannabis products report the concentration of two of the most abundant and widely studied phytocannabinoids, Δ^9^-tetrahydrocannabinol (THC) and cannabidiol (CBD). However, cannabis contains over 120 phytocannabinoids, most having unique pharmacological properties. One “minor” phytocannabinoid, cannabichromene (CBC), is commonly found in cannabis and ranges in published studies from 0.05 and 0.3% w/w [2–6].

In vitro pharmacological assays have revealed that CBC has multiple targets, including direct and indirect effects on the endocannabinoid system (ECS). CBC has low binding affinity and no appreciable activity at cannabinoid type 1 receptors (CB1), the receptor responsible for the intoxicating effects of THC [7]. However, CBC is a more efficacious cannabinoid type 2 receptor (CB2) agonist than THC, suggesting that CBC may be an effective anti-inflammatory agent [8]. CBC can inhibit endocannabinoid cellular reuptake and is a weak inhibitor of monacylglycerol lipase (MAGL), which may affect endocannabinoid tone [9, 10]. In addition to its action on the ECS, CBC is a potent activator and desensitizer of transient receptor potential (TRP) ankyrin 1-type (TRPA1) channels, indicating that CBC could be an antinociceptive agent [10, 11].

Preclinical research has identified several avenues of therapeutic potential that generally corroborate with in vitro pharmacological data. In vitro functional data show that CBC increases viability of adult neural progenitor cells and inhibited their differentiation into astroglia, suggesting that CBC may be a candidate for treating neuroinflammatory diseases [12]. In rodents, CBC has displayed anti-microbial, anti-inflammatory, analgesic, and anti-depressant-like activity [13–21].

Despite evidence from preclinical studies suggesting the therapeutic potential of CBC, its effects in humans have largely not been examined. One study detected CBC in plasma samples from medical cannabis patients who consumed CBD oil [22]. Another study assessing the efficacy and tolerability of a 1:20 THC:CBD medical cannabis product in children with treatment-resistant epileptic encephalopathy reported that the product contained 4% CBC by volume and described steady state trough levels of CBC [23]. However, acute post-dose pharmacokinetic (PK) data on CBC are critical to inform dosing schedules in future studies that evaluate the potential therapeutic effects of CBC in humans, and to understand how co-administration of multiple phytocannabinoids may impact the PK of each. The present pilot study examined the PK of CBC in human plasma from a study of a standardized oral medical cannabis product that contained 20 mg/mL CBD, 0.9 mg/mL THC, and 1.1 mg/mL CBC.

## Methods and materials

The parent study was a Phase 1, randomized, double-blind, placebo-controlled, multiple-dose trial in 43 healthy participants to assess the safety, tolerability, PK, and PD of Spectrum Yellow oil [24]. The study was conducted in accordance with consensus ethics principles, International Conference on Harmonization Good Clinical Practice guidelines, the Declaration of Helsinki, and local Australian laws and regulations. The protocol was approved by the Alfred Hospital Ethics Committee (Melbourne, Victoria, Australia). Written informed consent was obtained from each participant before any trial-related procedures were performed.

Spectrum Yellow oil (Tweed Inc., Canopy Growth Corporation, Smiths Falls, ON, Canada) is a cannabis-based product that is currently commercially available in Canada, Australia, United Kingdom, and Cayman Islands. Spectrum Yellow oil was made with supercritical carbon dioxide extracted cannabis resin in medium-chain triglyceride (MCT) oil. Analytical testing of the clinical batch detected 20 mg/mL CBD and 0.9 mg/mL THC, plus a total terpene concentration < 0.05%. Analytical testing of the clinical batch also detected the presence of CBC at a relatively high concentration (1.1 mg/mL), thus prompting the present subanalysis of the CBC time-concentration data. Analytical testing revealed that other cannabinoids were either below the reporting limit (< 0.50 ng/mL) or not detected.

Participants were randomly assigned to one of five groups in a 1:1:1:1:1 ratio: 120 mg CBD, 5.4 mg THC, and 6.6 mg CBC daily (Treatment A); 240 mg CBD, 10.8 mg THC, and 13.2 mg CBC daily (Treatment B); 360 mg CBD, 16.2 mg THC, and 19.8 mg CBC daily (Treatment C); 480 mg CBD, 21.6 mg THC, and 26.4 mg CBC daily (Treatment D); or placebo. Participants were confined to a residential research facility and received study medication twice daily, approximately every 12 h, after a standardized meal (e.g., for breakfast, 2 cups of cereal; 2 slices of toast; 2 servings of butter or margarine; 2 condiments; 250 mL of milk; 1 sugar sachet) for 6 days, plus a single dose in the morning of day 7. PK blood samples included in this analysis were collected prior to the morning dose and 1, 2, 4, 6, 8, and 12 h after the morning dose on day 1; prior to the morning dose and 1, 2, 4, 6, 8, 12, and 16 h after the morning dose on day 7; and 24, 32, 48, 72, 96, and 144 h after the day 7 morning dose. Immediately following collection, blood samples were placed on wet ice and centrifuged, and plasma was immediately frozen at −80 °C until shipment to the bioanalytical laboratory (iC42 Clinical Research and Development, University of Colorado, Aurora, CO, USA) on dry ice. Samples were stored at the bioanalytical laboratory at −80 °C.

CBC plasma concentrations were analyzed using a two-dimensional high-performance liquid chromatography–tandem mass spectrometry assay developed and validated by iC42 Clinical Research and Development [22], and study samples were analyzed in a CLIA (United States Clinical Laboratory Improvement Amendments)-certified laboratory environment accredited by the College of American Pathologists (Northfield, IL, USA). For details of the analytic method, please see Klawitter et al. [22]. The lower limit of quantification (LLoQ) of CBC was 0.78 ng/mL [22]; samples with concentrations below the LLoQ were treated as 0 in the analysis. Urinary excretion of CBC was not examined in this pilot study. PK parameters were calculated using non-compartmental analysis (Phoenix WinNonlin version 8.2., Certara, Princeton, NJ, USA). Statistical analysis was carried out using SPSS (version 27.0, IBM, Armonk, NY, USA).

## Results

Results on participant characteristics, safety and tolerability of Spectrum Yellow oil, and PK of CBD and THC in Spectrum Yellow oil are presented in the parent publication [24]. The overall conclusion was that Spectrum Yellow oil was safe and well-tolerated.

The majority of plasma samples on both days 1 and 7 had quantifiable concentrations of CBC except for Treatment A, where most samples were below the LLoQ (Table 1). Within each treatment group, there was notable variability between participants with respect to mean concentrations of CBC at each timepoint (Table 1). At an individual level, there appeared to be three different plasma concentration–time profiles of measured cannabinoids. Supplementary Material displays these three different profiles for participants in Treatment D on day 7: there was either low absorption of CBD and no absorption of either THC or CBC (Supplementary Material A), moderate absorption of CBD and CBC and no absorption of THC (Supplementary Material B), or high absorption of CBD, THC, and CBC (Supplementary Material C).

**Table 1:** Summary of plasma cannabichromene (CBC) concentrations (ng/mL) by treatment group

Timepoint	Treatment A^a^ (*n* = 8)			Treatment B^a^ (*n* = 8)			Treatment C^a^ (*n* = 8)			Treatment D^a^ (*n* = 8)	
	*n* BLoQ (*n*)	Mean (SD, CV [%])	*n* BLoQ (*n*)		Mean (SD, CV [%])	*n* BLoQ (*n*)		Mean (SD, CV [%])	*n* BLoQ (*n*)		Mean (SD, CV [%])
Day 1											
Predose	8 (8)	0 (0)	8 (8)		0 (0)	8 (8)		0 (0)	8 (8)		0 (0)
1 h	8 (8)	0 (0)	8 (8)		0 (0)	8 (8)		0 (0)	4 (8)		1.51 (1.90, 125.83)
2 h	7 (8)	0.27 (0.76, 281.48)	2 (8)		2.83 (3.48, 122.97)	5 (8)		1.43 (2.07, 144.76)	4 (8)		4.00 (4.44, 111.00)
4 h	6 (8)	0.69 (1.39, 201.45)	5 (8)		1.46 (2.37, 162.33)	3 (8)		2.86 (2.06, 72.03)	2 (8)		3.33 (2.95, 40.24)
6 h	8 (8)	0 (0)	7 (8)		0.27 (0.78, 288.89)	6 (8)		1.00 (1.94, 194.00)	5 (8)		1.34 (2.24, 167.16)
8 h	8 (8)	0 (0)	8 (8)		0 (0)	7 (8)		0.22 (0.62, 281.82)	7 (8)		0.35 (0.98, 280.00)
12 h	8 (8)	0 (0)	8 (8)		0 (0)	8 (8)		0 (0)	7 (8)		1.09 (3.07, 284.65)
Day 7											
Predose	7 (7)	0 (0)	8 (8)		0 (0)	7 (8)		0.22 (0.62, 281.82)	6 (8)		0.49 (0.92, 187.76)
1 h	5 (7)	0.69 (1.22, 176.81)	6 (8)		1.31 (2.75, 209.92)	7 (8)		0.36 (1.02, 283.33)	3 (8)		2.04 (2.22, 108.82)
2 h	6 (7)	0.38 (1.01, 265.79)	3 (8)		3.76 (5.41, 143.88)	4 (8)		3.03 (4.30, 141.91)	2 (8)		4.91 (4.47, 91.039)
4 h	7 (7)	0 (0)	2 (8)		2.44 (1.71, 70.08)	0 (8)		4.02 (1.66, 41.29)	1 (8)		4.47 (4.19, 93.74)
6 h	7 (7)	0 (0)	7 (8)		0.22 (0.63, 286.36)	4 (8)		1.59 (1.86, 116.98)	4 (8)		3.18 (5.56, 174.84)
8 h	7 (7)	0 (0)	8 (8)		0 (0)	5 (8)		0.78 (1.09, 1.67)	5 (8)		1.67 (2.65, 158.68)
12 h	7 (7)	0 (0)	8 (8)		0 (0)	8 (8)		0 (0)	5 (8)		1.00 (1.43, 143.00)

Timepoints are in relation to the morning dose. Concentrations that were below the lower limit of quantification were assigned as zero for analysis. *n* number, *BLoQ* below limit of quantification; lower limit of quantification of cannabichromene (CBC) is 0.78 ng/mL, *CV* coefficient of variation, *SD* standard deviation

^a^Treatment A: 120 mg CBD, 5.4 mg THC, and 6.6 mg CBC daily; Treatment B: 240 mg CBD, 10.8 mg THC, and 13.2 mg CBC daily; Treatment C: 360 mg CBD, 16.2 mg THC, and 19.8 mg CBC daily; and Treatment D: 480 mg CBD, 21.6 mg THC, and 26.4 mg CBC daily

Table 2 presents the summary plasma PK parameters for CBC. After a single dose on day 1, the maximum observed plasma concentration (*C*_max_) for CBC increased by 1.3- and 1.8-fold with each twofold increase in dose between Treatments A and B, and Treatments B and D; the median time to peak plasma concentration (*t*_max_) ranged 2.3–4.3 h across treatments. On days 2–7, almost all pre-dose concentrations of CBC were below the LLoQ in Treatments A, B, and C. On days 2–7, 3 of 8 participants in Treatment D had some quantifiable concentrations of CBC, but these were sporadic. Thus, steady-state concentration of CBC could not be calculated.

**Table 2:** Plasma pharmacokinetic parameters for cannabichromene (CBC)

Pharmacokinetic parameter (unit)	Treatment A^a^ (*n* = 8)		Treatment B^a^ (*n* = 8)		Treatment C^a^ (*n* = 8)		Treatment D^a^ (*n* = 8)	
	Day 1	Day 7	Day 1	Day 7	Day 1	Day 7	Day 1	Day 7
*C*_max_ (ng/mL)^b^	2.4 (43.6)^d^	2.8 (6.6)^e^	3.6 (60.1)^f^	4.0 (56.3)^g^	4.8 (20.9)^f^	4.2 (43.1)	6.6 (31.9)^h^	6.7 (42.6)^g^
*t*_max_ (h)^c^	3.2 (0.0–4.3)^d^	1.6 (1.3–2.0)^e^	2.3 (2.0–4.5)^f^	2.3 (2.0–4.3)^g^	4.3 (4.2–4.4)^f^	4.3 (2.3–4.3)	3.4 (2–12.3)^h^	2.3 (2.3–8.3)^g^
AUC_0–*t*_ (ng*h/mL)^b^	2.2 (75.2)^i^	2.3 (35.0)^e^	5.6 (83.9)^f^	5.9 (74.1)^g^	9.0 (41.4)^f^	9.9 (47.3)	17.6 (37.4)^h^	26.8 (31.6)^g^
AUC_0–12_ (h*ng/mL)^b^	-	5.7 (3.4)^e^	-	9.0 (46.3)^g^	-	13.6 (34.5)	-	29.5 (26.5)^g^
AUC_0–inf_ (h*ng/mL)^b^	NE	NE	NE	NE	NE	44.2 (6.9)^e^	NE	107.8 (-)^j^
% extrapolated (AUC_12–∞_/AUC_0–∞_)^b^	NE	NE	NE	NE	NE	18.22% (5.91%)^e^	NE	26.6% (-)^j^
CL/F (L/h)^b^	-	NE	-	NE	-	0.22 (17.4)^e^	-	0.12 (-)^j^

Concentrations that were below the lower limit of quantification were assigned as zero for analysis. *AUC*_*0–12*_ area under the plasma concentration–time curve from 0- to 12-h time point, *AUC*_*0–t*_ area under the plasma concentration–time curve from 0 to the last quantifiable concentration, *AUC*_*0–inf*_ area under the plasma concentration–time curve from 0 to infinity, *CL/F* oral clearance of drug from plasma, *C*_*max*_ maximum observed plasma concentration, *NE* not estimable, *t*_*max*_ time to reach *C*_*max*_

^a^Treatment A: 120 mg CBD, 5.4 mg THC, and 6.6 mg CBC daily; Treatment B: 240 mg CBD, 10.8 mg THC, and 13.2 mg CBC daily; Treatment C: 360 mg CBD, 16.2 mg THC, and 19.8 mg CBC daily; and Treatment D: 480 mg CBD, 21.6 mg THC, and 26.4 mg CBC daily

^b^Geometric mean (geometric CV%)

^c^Median (range)

^d^*n* = 4

^e^*n* = 2

^f^*n* = 5

^g^*n* = 7

^h^*n* = 6

^i^*n* = 3

^j^*n* = 1

Moderate accumulation was noted on day 7 after a week of twice-daily dosing of Spectrum Yellow oil, with the *C*_max_ increasing by 1.4- and 1.7-fold, and the area under the curve from time 0 to 12 h (AUC_0–12_) increasing by 1.6- and 3.3-fold with each twofold increase in dose of CBC between Treatments A and B, and Treatments B and D. On day 7, the median *t*_max_ of CBC ranged 1.6–4.3 h across treatments. The CL/F of CBC was only calculable for Treatments C and D, and ranged 0.12–0.22 L/h. On Days 1 and 7, while the dose of CBD was 18 times higher than that of CBC, the area under the curve from time 0 to last measurable concentration (AUC_0–*t*_) of CBD was only 6.6–9.8 fold higher than that of CBC (in Treatment D, where CBC was quantifiable in most samples); the dose of THC was similar to the dose of CBC, yet THC was quantifiable in fewer plasma samples than CBC.

**Fig. 1:**
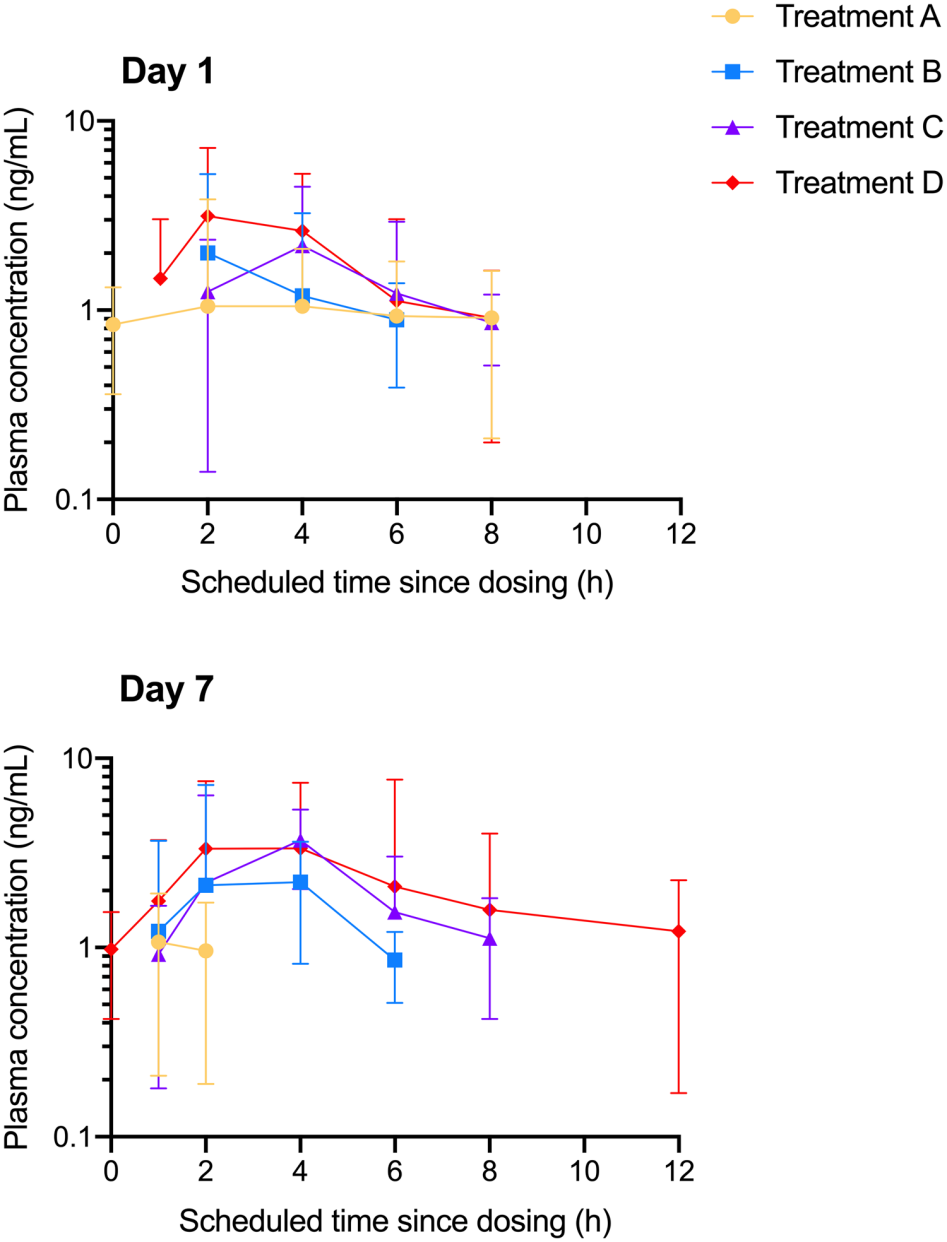
Geometric mean (± standard deviation) plasma concentration–time profiles for cannabichromene (CBC) in Spectrum Yellow oil on day 1 and day 7 for Treatment A: 120 mg CBD, 5.4 mg THC, and 6.6 mg CBC daily; Treatment B: 240 mg CBD, 10.8 mg THC, and 13.2 mg CBC daily; Treatment C: 360 mg CBD, 16.2 mg THC, and 19.8 mg CBC daily; and Treatment D: 480 mg CBD, 21.6 mg THC, and 26.4 mg CBC daily. Concentrations that were below the lower limit of quantification (0.78 ng/mL) were assigned as zero for analysis

Figure 1 shows the plasma concentration–time curves for CBC on days 1 and 7. These data suggested a dose-dependent increase in CBC plasma concentrations, although considerable variability was observed.

## Discussion

After a single dose, as well as a week of twice-daily dosing, of an oral cannabis product containing CBD, THC, and 1.1 mg/mL CBC, the *C*_max_ of CBC increased by 1.3–1.7-fold for each twofold increase in dose. Further, the observed CBC *t*_max_ ranged between 1.6 and 4.3 h, with between-participant variability in plasma concentrations of CBC.

A major finding of the parent study was that Spectrum Yellow oil was well-tolerated in healthy participants at daily doses up to 480 mg CBD and 20 mg THC [24]. Because Spectrum Yellow oil also contained 1.1 mg/mL CBC, it can thus be inferred that, in the presence of CBD and THC, CBC is well-tolerated up to daily doses of 26.4 mg. However, the present investigation was prompted by the observation that CBC was present in the clinical batch, and the studied doses (6.6–26.4 mg CBC daily) were not a priori based on doses found to be effective in published preclinical studies [13–21]. Future research evaluating the therapeutic potential of CBC may wish to study higher doses, and may need to collect data on safety at such doses.

Plasma concentrations of CBC were generally higher and more consistently quantifiable than those of CBD and THC at the same lower limit of quantification [24], relative to the dose administered. More specifically, while the dose of CBD was 18 times higher than the dose of CBC, the AUC_0–*t*_ of CBD was only 6.6–9.8 fold higher than the AUC_0–*t*_ of CBC (in Treatment D, where CBC was quantifiable in most samples); while the dose of THC was similar to the dose of CBC, THC was quantifiable in fewer plasma samples than CBC [24]. These data suggest that CBC may have preferential absorption over CBD or THC when administered together in Spectrum Yellow oil. It is interesting to note that clinical studies reported in the literature have produced conflicting findings on the interaction of different phytocannabinoids on each other’s pharmacokinetics. For example, in one study CBD delayed the time to reach peak plasma concentrations of THC [25], while other studies have shown that combining CBD with THC may lead to an increased peak concentration of plasma THC [26, 27], and others have shown no significant effect of CBD on the pharmacokinetics of THC [28]. These conflicting findings may partially be attributed to differences in cannabinoids and other compounds contained in the tested formulations.

As the presence of CBC appears common at low levels in CBD oils [22], it is important for future studies to compare the individual PK of CBD as well as CBC when administered as isolates to humans, to that of a product containing both CBD and CBC. Future studies should also investigate the effects of CBC on drug metabolism pathways, and should elucidate potential mechanisms underlying any interaction of multiple phytocannabinoids on pharmacokinetics, including rate and extent of absorption, competition for plasma protein binding, and inhibition/induction of cytochrome P450s that impact metabolism as well as of active drug transporters. Because this was a pilot study, a full pharmacokinetic study of CBC, including urinary excretion of CBC, is warranted.

## Conclusions

To our knowledge, these are the first data on the acute post-dose pharmacokinetics of the phytocannabinoid cannabichromene (CBC) in humans. At daily doses up to 26.4 mg, CBC in the presence of CBD and THC appears to be well-tolerated and is quantifiable in plasma in humans. Based on the ratio of administered phytocannabinoids to the amount measured in plasma, CBC may have preferential absorption over CBD and THC when administered together. CBC appears to be a viable target for further pharmacokinetic and therapeutic investigation.

## Supplementary information

Below is the link to the electronic supplementary material.

Supplementary file1 (TIFF 578 KB)

## Data Availability

The dataset analyzed during the current study is available from the corresponding author on reasonable request.
