# Supplementary figures and images for: Pharmacokinetics of cannabichromene in a medical cannabis product also containing cannabidiol and Δ9-tetrahydrocannabinol: a pilot study
Source: Eur J Clin Pharmacol. 2021 Oct 18;78(2):259–65. doi: 10.1007/s00228-021-03232-8 (PMC8748343; doi:10.1007/s00228-021-03232-8)

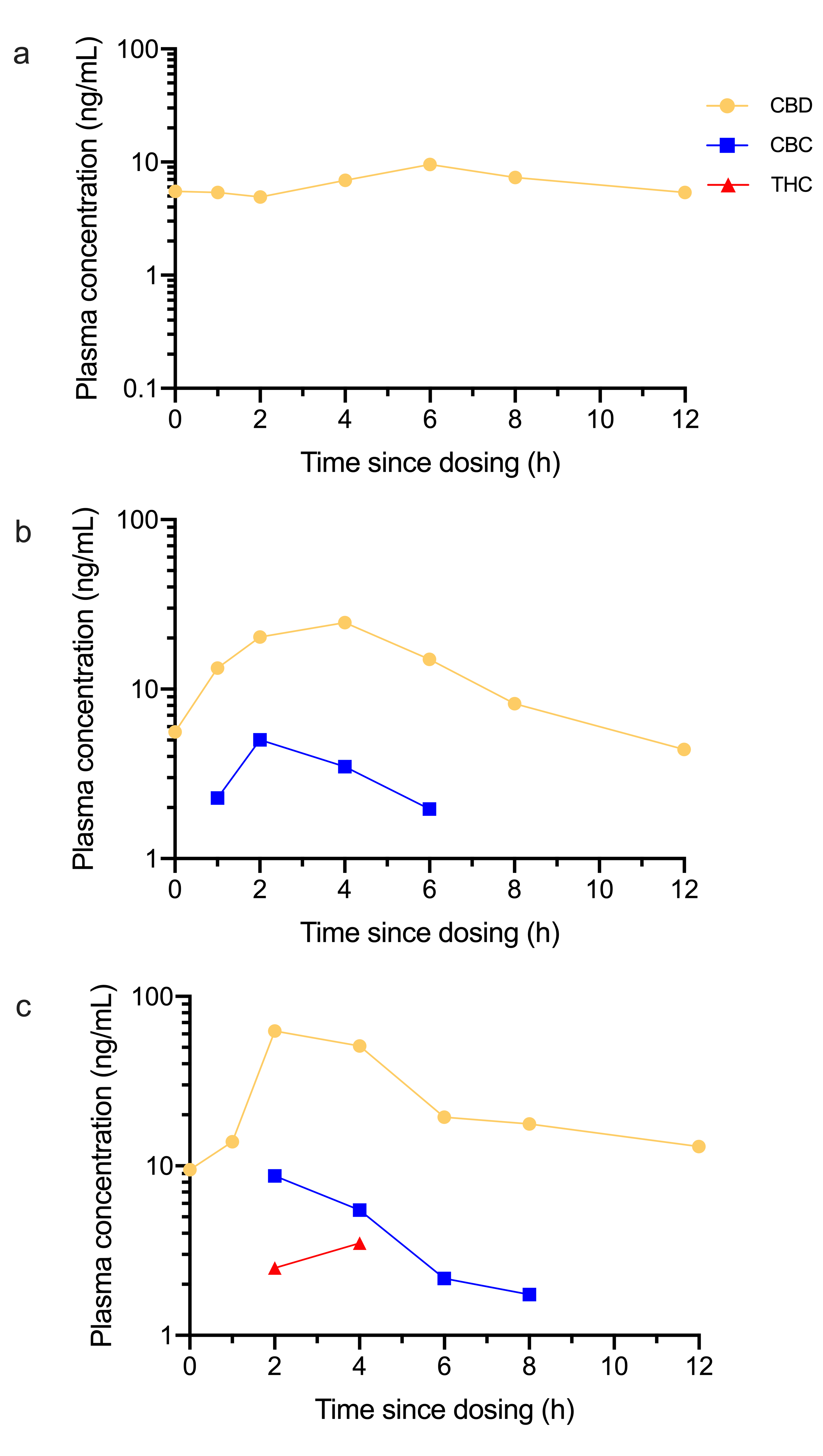

Supplement: Supplementary file 1 — Supplementary file1 (TIFF 578 KB) [file 228_2021_3232_MOESM1_ESM.tiff]
